# Isolation and Characterization of a Novel Pathogenic Strain of *Ehrlichia minasensis*

**DOI:** 10.3390/microorganisms7110528

**Published:** 2019-11-05

**Authors:** Daniel Moura de Aguiar, João Pessoa Araújo Junior, Luciano Nakazato, Emilie Bard, Lisandra Aguilar-Bultet, Fabien Vorimore, Vsevolod Leonidovich Popov, Edson Moleta Colodel, Alejandro Cabezas-Cruz

**Affiliations:** 1 Veterinary Hospital, Faculty of Veterinary Medicine, Federal University of Mato Grosso State (UFMT), 78060-900 Cuiabá, Brazil; lucnaka@gmail.com (L.N.); moleta@ufmt.br (E.M.C.); 2 Biotechnology Institute (IBTEC), São Paulo State University (UNESP), 18607-440 Botucatu, Brazil; joao.pessoa@unesp.br; 3 EPIA, INRA, VetAgro Sup, 63122 Saint Genès Champanelle, France; emilie.bard@inra.fr; 4 Department of Infectious Diseases and Hospital Epidemiology, University Hospital Basel, 4031 Basel, Switzerland; lisandritta@gmail.com; 5 University Paris-Est, Anses, Animal Health Laboratory, Bacterial Zoonoses Unit, 94706 Maisons-Alfort, France; fabien.vorimore@anses.fr; 6 Department of Pathology, University of Texas Medical Branch at Galveston, Galveston, TX 77555, USA; vpopov@utmb.edu; 7 UMR BIPAR, INRA, ANSES, Ecole Nationale Vétérinaire d’Alfort, Université Paris-Est, 94700 Maisons-Alfort, France

**Keywords:** anaplasmataceae, *Ehrlichia minasensis*, bovine ehrlichiosis, DH82, transmission electron microscopy, genome

## Abstract

The genus *Ehrlichia* is composed of tick-borne obligate intracellular gram-negative alphaproteobacteria of the family Anaplasmataceae. *Ehrlichia* includes important pathogens affecting canids (*E. canis*, *E. chaffeensis*, and *E. ewingii*), rodents (*E. muris*), and ruminants (*E. ruminantium*). *Ehrlichia* *minasensis*, an *Ehrlichia* closely related to *E. canis*, was initially reported in Canada and Brazil. This bacterium has now been reported in Pakistan, Malaysia, China, Ethiopia, South Africa, and the Mediterranean island of Corsica, suggesting that *E. minasensis* has a wide geographical distribution. Previously, *E. minasensis* was found to cause clinical ehrlichiosis in an experimentally infected calf. The type strain *E. minasensis* UFMG-EV was successfully isolated from *Rhipicephalus microplus* ticks and propagated in the tick embryonic cell line of *Ixodes scapularis* (IDE8). However, the isolation and propagation of *E. minasensis* strains from cattle has remained elusive. In this study, the *E. minasensis* strain Cuiabá was isolated from an eight-month-old male calf of Holstein breed that was naturally infected with the bacterium. The calf presented clinical signs and hematological parameters of bovine ehrlichiosis. The in vitro culture of the agent was established in the canine cell line DH82. Ehrlichial morulae were observed using light and electron microscopy within DH82 cells. Total DNA was extracted, and the full genome of the *E. minasensis* strain Cuiabá was sequenced. A core-genome-based phylogenetic tree of *Ehrlichia* spp. and *Anaplasma* spp. confirmed that *E. minasensis* is a sister taxa of *E. canis*. A comparison of functional categories among *Ehrlichia* showed that *E. minasensis* has significantly less genes in the ‘clustering-based subsystems’ category, which includes functionally coupled genes for which the functional attributes are not well understood. Results strongly suggest that *E. minasensis* is a novel pathogen infecting cattle. The epidemiology of this *Ehrlichia* deserves further attention because these bacteria could be an overlooked cause of tick-borne bovine ehrlichiosis, with a wide distribution.

## 1. Introduction

The genus *Ehrlichia* belongs to the family Anaplasmataceae and consists of six recognized species of bacteria: *E. canis*, *E. chaffeensis*, *E. muris*, *E. ewingii*, *E. ruminantium*, and *E. minasensis* [1,2]. Prior to 2010, ehrlichiosis in bovines was mostly associated with infection by *E. ruminantium*, a bacterium present in the African continent and in some regions of the Caribbean [3,4]. However, in the early 2010s, an ehrlichial species closely related to *E. canis* was reported to be infecting cattle and deer in British Columbia, Canada [5,6]. The isolation, in vitro culture, and molecular characterization of this agent was achieved later, when the same bacteria species was isolated from a partially engorged *Rhipicephalus microplus* female tick collected in Minas Gerais, Brazil [7,8]. In 2014, *E. minasensis* was isolated from dairy and beef cattle in Midwestern Brazil, and it was found to cause clinical ehrlichiosis in an experimentally infected calf [9].

In addition to *R. microplus* and other species of the genus *Rhipicephalus*, *R. appendiculatus*, *R. eversti* eversti, *R. sanguineus* s.l., and *R. bursa* [7,10,11], *E. minasensis* has also been identified in *Amblyomma* [11], *Hyalomma* [12,13], and *Haemaphysalis* [14], suggesting that several tick species could vector this bacterium. *E. minasensis* has a wide distribution; it has been reported in Canada [5,6], Brazil [7,9], Pakistan [13], Malaysia [15], China [14], Ethiopia [16], South Africa [11], and the Mediterranean island of Corsica [12]. These findings suggest that *E. minasensis* may be transmitted by more than one tick species which explain the wide geographical distribution of this bacterium [17].

In a previous report [9], the isolation of *E. minasensis* from a bovine blood sample and the in vitro culture of this bacterium in canine macrophage cell line DH82 were attempted. However, despite successful isolation, the propagation of the bacterium could not be established for more than one month [9]. In this study, we describe, for the first time, the isolation of *E. minasensis* from a blood sample of a naturally infected bovine. The successful propagation of *E. minasensis* was achieved in DH82 cells which were used for morphologic and genetic characterization of this bacterium by electron microscopy and genome sequencing, respectively. This study provides a comprehensive characterization of the *E. minasensis* strain Cuiabá and seeks to contribute to the better understanding of this pathogen.

## 2. Material and Methods

### 2.1. Animal and Sample Collection

An eight-month-old male calf of Holstein breed was admitted in May 2015 to the Veterinary Hospital of the Faculty of Veterinary Medicine of the Federal University of Mato Grosso State (UFMT). The calf belonged to a dairy herd infected with *E. minasensis* [9,10]. The farm is located at Santo Antônio do Leverger municipality, a small town 20 km distance from Cuiabá, the capital of Mato Grosso State, where practical classes of veterinary students of UFMT were carried out. A whole-blood sample was collected from the jugular vein in EDTA tubes, and hematological analyses were performed and compared with reference values previously reported [18].

### 2.2. DNA Extraction and Polymerase Chain Reaction

Genomic DNA was extracted from blood and DH82 cell culture, using the Wizard Genomic DNA purification Kit (Promega, Madison, WI, USA) according to the manufacturer’s instructions. For molecular diagnosis, a heminested PCR protocol was used to amplify a fragment of the *Ehrlichia* gene *dsb*. The first PCR reaction targeted 401-bp (primers: Dsb-330 5′ GATGATGTTTGAAGATATSAAACAAAT 3′ and Dsb-720 5′ CTATTTTACTTCTTAAAGTTGATAWATC 3′), and the second reaction targeted 349-bp (primers: Dsb-380 5′ ATTTTTAGRGATTTTCCAATACTTGG 3′ and Dsb-720), as previously reported [9]. Amplification was carried out by following a protocol involving 1.25 U GoTaq™ Hot Start Polymerase (Promega, Madison, WI, USA) and 10 pmol/µL of each primer, according the manufacturer’s instructions. DNA of *E. canis* strain Cuiabá#1 and template free reactions were used as positive () and negative controls of the PCR reactions. Amplicons were visualized by agarose gel electrophoresis (1.5%).

### 2.3. In Vitro Culture of Ehrlichia

Blood samples were processed for isolation of *Ehrlichia* in cell culture. To this end, the blood was aseptically collected in EDTA vacuum tubes and transported to the laboratory for mononuclear cells (MNCs) isolation, using Histopaque 1083 (Sigma-Aldrich, St. Luis, MO, USA), as previously described [19]. Cultures were initiated by seeding the MNCs in a 25 cm^2^ culture flask containing Dulbecco’s Modified Eagle’s medium (DMEM, Sigma-Aldrich, St Louis, MO, USA) and supplemented with 20% iron-fortified Bovine Calf Serum (BCS, Sigma-Aldrich, St Louis, MO, USA). The culture flask was kept at 37 °C and 5% CO_2_. Every two days, 1/5 of the primary culture medium was collected for cytologic evaluation, using Romanowsky-stained smears (NewProv, Pinhais, PR, Brazil), and fresh medium was added. After 96 hours (h) of incubation, DH82 cells were added to the primary culture of MNCs, and the supplementation of DMEM was reduced to 5% iron-fortified BCS. The culture was kept at 37 °C and 5% CO_2_. Every seven days, samples were collected for cytologic evaluation and PCR. Stocks of infected DH82 cells were resuspended in cell-freezing medium (Sigma-Aldrich, St Louis, MO, USA) and frozen at −156 °C in liquid nitrogen.

### 2.4. Transmission Electron Microscopy

The samples for transmission electron microscopy were prepared following protocols previously described [20]. Briefly, the monolayers of DH82 cells infected with *E. minasensis* were fixed in 2.5% paraformaldehyde and 0.1% glutaraldehyde in 0.05 M cacodylate buffer, pH 7.3, to which 0.01% trinitrophenol and 0.03% CaCl_2_ were added. After fixation, the cells were washed with 0.1 M cacodylate buffer, scraped off the flasks, and pelleted. The pellets were post-fixed in 1% OsO_4_ in 0.1 M of cacodylate buffer, en bloc stained with 2% aqueous uranyl acetate in 0.1 M maleate buffer, dehydrated in ethanol, and embedded in Poly/Bed 812 (Polysciences, Warrington, PA, USA). Ultrathin sections were cut on Reichert-Leica Ultracut S ultramicrotome, stained with lead citrate, and examined in a Philips 201 or CM-100 electron microscope at 60 kV.

### 2.5. Genome Sequencing

The genome of the *E. minasensis* strain Cuiabá was recently sequenced, annotated, and announced [21]. Briefly, DNA was extracted from DH82 cells infected with *E. minasensis*, and a Nextera XT library was prepared (Illumina, San Diego, CA, United States). Sequencing was performed using the sequencer NextSeq 550 System (Illumina, San Diego, United States). A total of 37 million reads passed the quality filter and were merged and assembled. Genome annotation was run with Prokka 1.14 [22]. The raw sequence reads were submitted to NCBI SRA and are available under the accession number PRJNA478569. The whole-genome shotgun project of the *E. minasensis* strain Cuiabá was deposited in DDBJ/EMBL/GenBank and is available under the accession number QOHL00000000. Genome assembly data are available under the accession number GCA_004181775.

### 2.6. Core-Genome-Based Phylogenetic Tree

Genome assemblies of *E. canis* (2 strains), *E. chaffeensis* (9 strains), *E. muris* (2 strains), *E. ruminantium* (10 strains), and *E. minasensis* (2 strains) available in the refseq database of NCBI were downloaded in fasta format. Genome accession numbers of the strains used are available in Supplementary Table S1. Three *Anaplasma* genomes (i.e., *A. marginale*-SAMN02603338, *A. centrale-*SAMN02604279 and *A. phagocytophilum*-SAMN02585077) were used as outgroups in the core-genome-based phylogenetic tree. The coding sequences of the genomes were used for ortholog genes identification, using the OrthoMCL pipeline [23]. The nucleotide sequences of all the ortholog genes identified in the 28 bacterial genomes were aligned using MACSE [24]. The final alignment included 97,908 nucleotide positions of 125 protein-coding genes present in the core-genome of the selected bacteria. A maximum-likelihood phylogenetic tree was then built by using the GTR model and the software PhyML 3.0 [25]. Reliability of internal branches was assessed by using the approximate likelihood ratio test (aLRT) implemented in Seaview [26].

### 2.7. Bacterial Pan-Genome Profiles

Gene clusters of *Anaplasma* and *Ehrlichia* genomes (see Section 2.6) were predicted with OrthoMCL [23], using best-matching gene similarities with an *E*-value ≤ 1 ×10^−5^. The number of gene clusters was plotted against the number of genomes, and the resulting rarefaction curves were used to visualize the pan-genome and core-genome of *Anaplasma* and *Ehrlichia*. Rarefaction curves were built by using the settings recommended for datasets larger than 15 genomes. Pan-genome and core-genome profiles were calculated with the software PanGP [27].

### 2.8. Genome Comparative Analysis

The functional annotation of *E. minasensis* Cuiabá genome (SAMN09519635) was compared to that of other 9 genomes of the Order Rickettsiales: *A. central* Israel (SAMN02604279), *A. marginale* Florida-1 (SAMN02469629), *A. phagocytophilum* HZ2 (SAMN02604172), *E. canis* Jake (SAMN02598261), *E. chaffeensis* Arkansas (SAMN02604010), *E. muris* AS145 (SAMN02641637), *E. ruminantium* Welgevonden (SAMEA1705918), *Rickettsia conorii* Malish (SAMN02603141), and *Rickettsia prowazekii* Rp22 (SAMN02604077). All genomes were functionally annotated, using PATRIC [28], a web-based tool that uses RAST [29], which assigns gene functions and groups them according to different categories: superclass, class, subclass, and subsystems. The two-proportion test, implemented in R (prop.test function), was used to detect differences between the functional categories of *E. minasensis* and the other genomes, with a 95% confidence interval [30].

## 3. Results

### 3.1. Natural Infection of E. minasensis in a Calf

An eight-month-old male calf of Holstein breed, from a dairy farm, presented fever, depression, and lethargy. The farm had a history of chronic *R. microplus* infestation and *E. minasensis* infection [9,10]. The animal was sent to the veterinary hospital, and routine examinations were performed by members of the faculty of veterinary medicine. Among the laboratory tests performed, the hematological analysis revealed abnormalities, including anemia (4.7 × 10^6^ cells/mm^3^, 5.5 g/dl hemoglobin, 18% packed cell volume, and 38 femtoliters (fl) of mean corpuscular volume), leukopenia (3.7 × 10^3^ cells/ mm^3^), and thrombocytopenia (124 × 10^3^ platelets /mm^3^). Blood infection with *Ehrlichia* was confirmed by PCR amplification of a fragment of the *Ehrlichia* gene *dsb*.

### 3.2. Isolation and In Vitro Culture of E. minasensis

**Figure 1:**
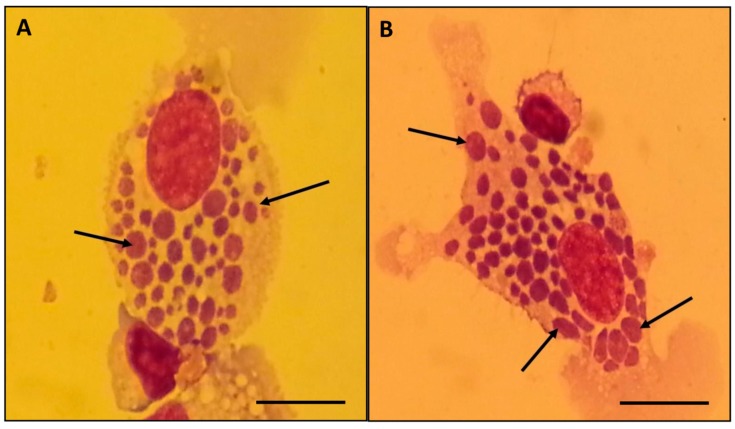
Romanowsky-stained smears showing large morulae of *E. minasensis* (arrows) in the cytoplasm of calf monocytes. Images (**A**) and (**B**) each show a different monocyte heavily infected with multiple morulae of *E. minasensis*. Magnification, 1000×. Bar = 10 µm.

MNCs were isolated from the peripheral blood of the *Ehrlichia*-infected calf and seeded in DMEM supplemented with 20% BCS. After 72 h of incubation, irregular clusters of ehrlichial morulae were observed in monocytes of the primary culture (Figure 1).

**Figure 2:**
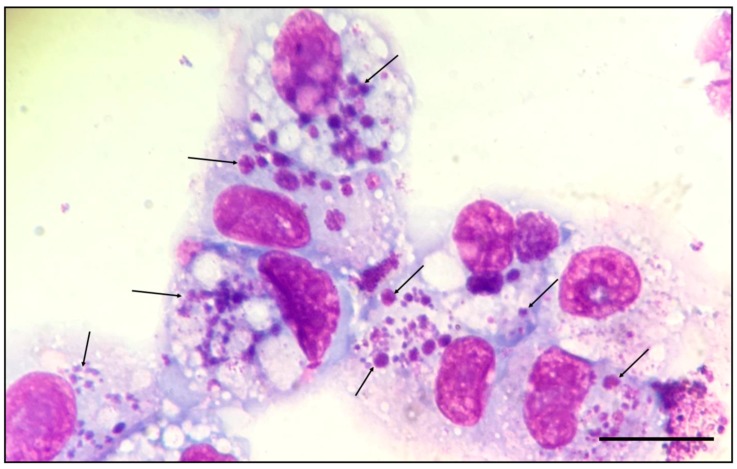
Romanowsky-stained stained smear showing morulae of *E. minasensis* (arrows) in the cytoplasm of DH82 cells. Magnification, 1000×. Bar = 10 µm.

One day after the ehrlichial morulae were observed in the primary culture, DH82 cells were added in the primary culture. Seven days later, ehrlichial morulae were observed within the cytoplasm of 10% of the DH82 cells (Figure 2).

After one month in culture, 90%–100% of DH82 cells were infected with *E. minasensis*. Subsequently, infected DH82 cells were subcultured into uninfected DH82 cells, and the infection reached 90%–100% within 10 to 14 days. Frozen stocks of *Ehrlichia*-infected DH82 cells were thawed, and their infectivity was tested. High infectivity was observed when the thawed material was added to uninfected DH82 monolayers. The *E. minasensis* isolate, designated as the Cuiabá strain, was deposited in the *Ehrlichial* collection of the Laboratory of Virology and Rickettsiosis of the Faculty of Veterinary Medicine of UFMT, where it is available upon request.

### 3.3. Electron Microscopy Characterization

**Figure 3:**
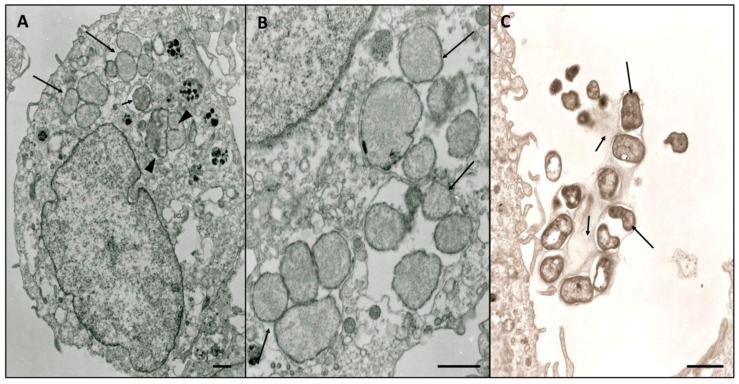
Electron micrograph of *E. minasensis* in DH82 cells. (**A**) Morulae were observed to contain reticulate cells (RC, large arrow), densely packed unique cell (small arrow), and cells dividing by unequal fission (head arrow). (**B**) RC bodies inside membrane-lined vacuoles were bound by an inner plasma membrane and an outer cell wall (arrow). (**C**) Dense-cored cells DC microorganisms (large arrow) surrounded by cellular plasmalemma (small arrow) after rupture of the host cell membrane. Bar = 1.0 µm.

*E. minasensis* developed within multiple parasitophorous vacuoles in the cytoplasm of DH82 cells (Figure 3A). The number of morulae per cell ranged from 1 to 16. The average number of microorganisms per morulae was five. Homogeneous populations of reticulate cells (RCs) (Figure 3B) and dense-cored cells (DCs) (Figure 3C) were observed.

RCs were small, oval and surrounded by two limiting membranes: an outer cell-wall membrane and inner cytoplasmic membrane. DCs were round with irregular or pleomorphic shape. Ehrlichial DCs were observed after the rupture of the host cell membrane. DCs were surrounded by the plasmalemma (Figure 3C). The diameter of the RCs ranged from 0.7 to 2.0 × 0.6 to 1.2 µm, while the DCs ranged from 0.5–1.5 × 0.4 to 1.0 µm.

### 3.4. Genome Properties and Phylogenetic Analysis

*E. minasensis* Cuiabá genome consists of 1,335,478bases, with an N50 of 694,769 and 29.5% G+C content, and revealed the presence of 1270 ORFs, including coding sequences (CDS, 1231) and RNA genes (3 rRNAs and 36 tRNAs). Comparison of general features of the *E. minasensis* Cuiabá genome with those of other *Anaplasma*, *Ehrlichia*, and *Rickettsia* species revealed similarities, but also differences between these genomes (Table 1).

**Figure 4:**
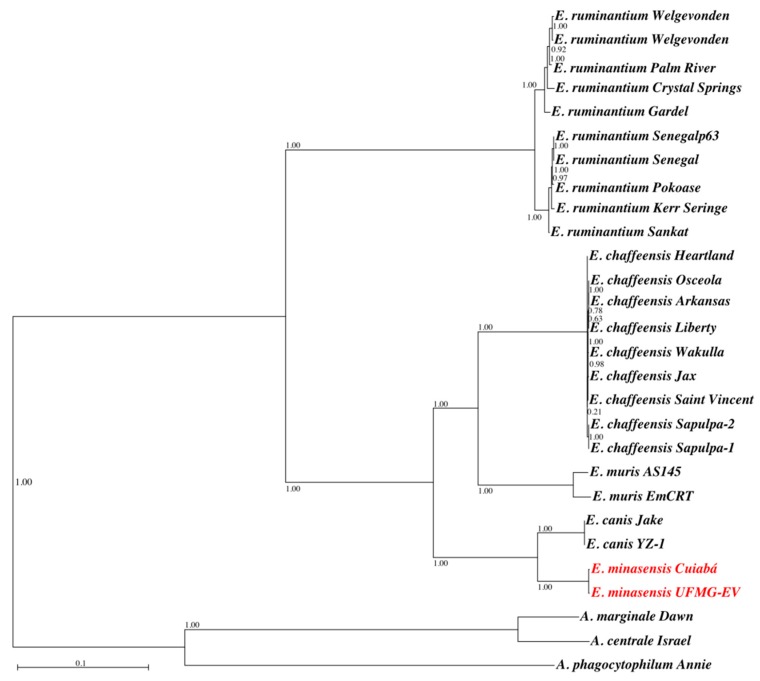
Core-genome-based phylogenetic tree of *Ehrlichia*. A maximum-likelihood phylogenetic tree was built, using the GTR model and the software PhyML. The final alignment included the nucleotide sequences of 125 protein-coding genes present in the core-genome of the selected bacteria. The genomes of *A. marginale*, *A. centrale*, and *A. phagocytophilum* were included as outgroups. The two strains of *E. minasensis* are highlighted in red. Values on internal branches represent the statistical support of the topology calculated by aLRT.

Rarefaction curves built with gene clusters of 3 *Anaplasma* and 25 *Ehrlichia* genomes show that the pan-genome and core-genome of these bacteria have approximately 3000 and 400 genes, respectively (Supplementary Figure S1). A maximum-likelihood phylogenetic tree was built based on the alignment of 97,908 nucleotide positions of 125 CDS of the core-genome of *Anaplasma*, *Ehrlichia*, and *Rickettsia* (Figure 4).

The phylogenetic tree shows that *E. minasensis* strains Cuiabá and UFMG-EV cluster together and are both closely related to *E. canis* (Figure 4).

### 3.5. Genome Comparison Analysis

Genome functional annotations of *E. minasensis* strain Cuiabá were compared to those of other species of *Anaplasma*, *Ehrlichia*, and *Rickettsia* (Table 2).

Major functional categories were distributed across 20 classes, and no significant differences were found in the amount of genes involved in carbohydrate metabolism, DNA processing, energy and precursor metabolites generation, membrane transport, metabolite damage and its repair or mitigation, phosphate metabolism, prokaryotic cell type differentiation, protein fate (i.e., folding, modification, targeting, and degradation), and miscellaneous. This suggests that the amount of functions in these categories is highly conserved among the selected Rickettsiales. For the rest of the 11 categories, differences were found between *E. minasensis* and at least one of the other bacteria. The only significant difference between *E. minasensis* and *E. canis*, *E. chaffeensis*, and *E. muris* was in the category clustering-based subsystems in which *E. minasensis* showed a significantly lower number of genes (i.e., four) compared with these three *Ehrlichia* (i.e., between 16 and 17). A pairwise comparison between *E. minasensis* and *E. ruminantium* revealed that *E. minasensis* has a significant reduction in genes of clustering-based subsystems (i.e., 16 in *E. ruminantium* and four in *E. minasensis*) and respiration (i.e., 103 in *E. ruminantium* and 94 in *E. minasensis*). Concerning *Anaplasma* and *Rickettsia*, *E. minasensis* genome functional annotation had significant differences with *A. centrale* (four categories), *A. marginale* (one category), *A. phagocytophilum* (two categories), *R. conorii* (seven categories), and *R. prowazekii* (five categories) (Table 2).

## 4. Discussion

This paper reports the isolation of *E. minasensis* from a naturally infected calf from the midwestern region of Brazil. *E. minasensis* was previously isolated from *R. microplus* ticks [2,7], and it was already known that this bacterium was able to infect cattle and, after experimental infection, produce clinical manifestations associated with ehrlichiosis [9]. However, this is the first report of an *E. minasensis* strain isolated directly from a naturally infected animal with clinical signs of ehrlichiosis. In addition to *E. minasensis*, the other *Ehrlichia* species that infects ruminants is *E. ruminantium*, which is the causative agent of heartwater or cowdriosis mainly in African bovines [3]. While *E. ruminantium* is a recognized cause of ehrlichiosis in bovines, the ability of *E. minasensis* to cause this disease has been overlooked [17].

The clinical signs and blood parameters of the naturally infected calf described in this study concurred with those previously described for a calf experimentally infected with *E. minasensis* [9]. The clinical signs caused by virulent *E. ruminantium* strains include elevated temperature, loss of appetite, heavy breathing, hanging head, depression, exaggerated blinking and chewing movements, anorexia, hyperesthesia, lacrimation, convulsions, and death [3]. As most of the clinical signs produced by *E. minasensis* and *E. ruminantium* would not individually constitute a definitive diagnosis, and there is also an overlap of some signs (e.g., fever and depression), the postmortem examination plays a crucial role in the differential diagnosis of these two *Ehrlichia*. As the macroscopic lesions produced by *E. ruminantium* are different to those produced by *E. minasensis*, a differential postmortem diagnosis can be established for the infection with these pathogens. Common lesions caused by *E. ruminantium* include hydropericardium, hydrothorax, and increased vascular permeability, which are associated with edema of the lungs, brain, and other organs [3]. In contrast, the major macroscopic lesion observed in animals experimentally infected with *E. minasensis* was enlargement and diffuse swelling of mesenteric lymph nodes, and no pathologic changes were observed in the lung, spleen, kidney, stomach, and brain [9]. Histopathological examination is also useful, as *E. ruminantium* morulae are found in the cytoplasm of endothelial cells, whereas *E. minasensis* morulae are found mostly in the cytoplasm of MNCs of peripheral blood. The clinical signs caused by *E. minasensis* in bovines appear more similar to those of canine ehrlichiosis caused by *E. canis* in dogs [9,31], in which a chronic development of the disease with an asymptomatic phase is typical [32]. Experimental infection with *E. minasensis* was also reported in splenectomized calves, using the BC strain in Canada; however no clinical manifestations were observed, probably due to the use of a nonpathogenic *E. minasensis* strain [5,33].

In order to ensure the transfer of the microorganisms from the host MNCs to the cell line DH82, two major procedures were followed. First, the primary culture of bovine MNCs was incubated for 96 h, in order to increase the infection rates of *Ehrlichia*. Second, after the visualization of a large number of *Ehrlichia* morulae, uninfected DH82 cells were added to the primary culture. This strategy enabled the isolation of *E. minasensis* into DH82 cells. Earlier isolation of *E. minasensis* was achieved successfully in IDE8 cells, which were used as the source of infection for the DH82 cells [8]. In this study, it was possible to isolate *E. minasensis* in DH82 cells directly from infected bovine cells.

Ultrastructural characterization of *E. minasensis* Cuiabá showed that this isolate has typical morphological features of the genus *Ehrlichia*. Among these features are the presence of pleomorphic RC and DC cells surrounded by two membranes (the cell membrane and cytoplasmic membrane), the location of microorganisms within membrane-bound compartments (morulae), and the size of the cells which are comparable with those of other *Ehrlichia* cultured in mammalian cells [20,34]. In one of the images (Figure 3C), it was possible to observe the DC cells leaving the host cell and moving to the extracellular environment, after the rupture of the morulae.

In agreement with previous molecular analysis using the bacterial genes 16S rRNA, *groEL*, *dsb*, *gltA*, and *trp36* [7,9], the core-genome-based phylogenetic tree confirmed that *E. minasensis* is a sister taxa of the dog pathogen *E. canis*, and, despite infecting the same host, *E. minasensis* and *E. ruminantium* are distantly related pathogens [2]. Previous molecular evolution analysis based in the gene *trp36* suggested that *E. minasensis* evolved from highly variable strains of *E. canis* [35]. Genome comparison analysis showed that the *E. minasensis* Cuiabá genome is bigger and has more CDS than that of *E. canis*. This suggests that diversification and evolution of host-shift (i.e., from dogs to cattle, [35]) in *E. minasensis* might had been related to genome expansion and the acquisition of new genes associated with virulence and infection in cattle. The genome expansion could also be explained by an increase in the junk DNA sequence. However, the discrimination between the factors explaining the genome expansion in *E. minasensis*, compared with *E. canis*, is out of the scope of this manuscript. A high-resolution comparison of the genomes of *E. canis* and *E. minasensis* will contribute to understanding the host-shift in *Ehrlichia*. The rationale behind this idea is that pathogen adaptation to new hosts is usually associated with changes in the genome [36]. Indeed, recent studies have investigated host-shifts, with an emphasis on genetic changes, such as mutations, hybridizations, chromosomal reorganizations, or horizontal gene transfer events, involved in host-shift genetics [36]. Host-shift can involve multiple factors; and certainly, other factors influencing host-shift such as phenotypic plasticity and/or cryptic genetic variation in the pathogen population should also be considered. In this preliminary study, the only functional category found to be significantly different between *E. minasensis* Cuiabá and *E. canis* was clustering-based subsystems in which *E. minasensis* has significantly less genes than *E. canis* and the other *Ehrlichia*. Clustering-based subsystems include functionally coupled genes (genes found proximal to each other in the genomes of diverse taxa) for which the functional attributes are not well understood. However, it is noteworthy that, despite the fact that the differences were not significant, *E. minasensis* has more genes than *E. canis*, in other important categories, such as membrane transport; cell cycle, division, and death; protein synthesis; RNA processing; and stress response, defense, and virulence. It is also important to mention that a single gene could be functionally relevant and provide an adaptive advantage in new environments.

## 5. Conclusions

The isolation and characterization of *E. minasensis* Cuiabá from a naturally infected bovine, together with the previous report of experimental infection of this bacterium in the same type of host [9], confirms that *E. minasensis* is a novel pathogen that causes bovine ehrlichiosis in Brazil. However, further studies are required to elucidate the potential pathogenicity of *E. minasensis* strains found in countries other than Brazil. In addition to adding genetic information to the study of the Anaplasmataceae family, the access to the *E. minasensis* genome will help elucidating the genetic basis of host-shift in *Ehrlichia*. The identification of major antigenic proteins of *E. minasensis* will be a crucial step towards the development of diagnostic tests and vaccines to control infection by thispathogen.

## Figures and Tables

**Table 1:** Genome properties.

Features *	Bacteria									
	EMI	ECA	ECH	EMU	ER	AC	AM	AP	RC	RP
Size (bp)	1,335,478	1,315,030	1,176,248	1,196,717	1,516,355	1,206,806	1,136,981	1,477,581	1,268,755	1,111,612
GC (%)	29.5	29.0	30.1	29.7	27.5	50.0	49.8	42.0	32.4	29.0
tRNA	36	36	37	37	36	37	35	37	33	33
rRNA	3	2	2	2	2	2	3	3	2	2
total ORFs	1270	1068	1032	1055	1056	1194	1359	1501	1637	960
PATRIC CDS	1231	1030	992	992	1015	1153	1321	1447	1578	919
Contigs	55	1	1	1	1	1	204	1	1	1

* Features based on PATRIC annotations of one strain of each species. Abbreviations as follow: EMI, *E. minasensis* Cuiabá; ECA, *E. canis*; ECH, *E. chaffeensis*; EMU, *E. muris*; ER, *E. ruminantium*; AC, *A. centrale*; AM, *A. marginale*; AP, *A. phagocytophilum*; RC, *R. conorii*; RP, *R. prowazekii*. Genome accessions are available in material and methods (Section 2.8).

**Table 2:** Comparison of genome functional features.

Class	EMI	ECA	ECH	EMU	ER	AC	AM	AP	RC	RP
Amino Acids and Derivatives	0.034 (42)	0.036 (37)	0.037 (37)	0.041 (41)	0.042 (43)	0.021 (24)	0.030 (39)	0.005*** (7)	0.015** (24)	0.026 (24)
Carbohydrates	0.002 (2)	0.002 (2)	0.002 (2)	0.002 (2)	0.002 (2)	0.002 (2)	0.002 (2)	0.001 (2)	0.001 (2)	0.002 (2)
Cell Cycle, Cell Division and Death	0.011 (13)	0.006 (6)	0.006 (6)	0.006 (6)	0.006 (6)	0.029** (33)	0.036*** (47)	0.004 (6)	0.029** (45)	0.032*** (29)
Cell Envelope, Capsule and Slime layer	0.002 (2)	0.002 (2)	0.002 (2)	0.002 (2)	0.002 (2)	0.004 (5)	0.004 (5)	0.001 (2)	0.008* (13)	0.014** (13)
Clustering-based subsystems	0.003 (4)	0.0165** (17)	0.017** (17)	0.016** (16)	0.016** (16)	0.015** (17)	0.004 (5)	0.007 (10)	0.003 (4)	0.004 (4)
Cofactors, Vitamins, Prosthetic Groups	0.084 (104)	0.098 (101)	0.102 (101)	0.104 (103)	0.101 (103)	0.087 (100)	0.090 (119)	0.077 (111)	0.042*** (66)	0.069 (63)
DNA Processing	0.032 (40)	0.039 (40)	0.040 (40)	0.042 (42)	0.044 (45)	0.042 (49)	0.048 (63)	0.036 (52)	0.034 (54)	0.038 (35)
Energy and Precursor Metabolites Generation	0.037 (46)	0.045 (46)	0.046 (46)	0.050 (50)	0.046 (47)	0.040 (46)	0.039 (52)	0.037 (53)	0.027 (42)	0.041 (38)
Fatty Acids, Lipids, and Isoprenoids	0.037 (45)	0.043 (44)	0.044 (44)	0.045 (45)	0.041 (42)	0.036 (42)	0.028 (37)	0.029 (42)	0.018** (28)	0.030 (28)
Membrane Transport	0.044 (54)	0.046 (47)	0.047 (47)	0.049 (49)	0.054 (55)	0.056 (65)	0.058 (77)	0.039 (57)	0.037 (59)	0.063 (58)
Metabolite damage and its repair or mitigation	0.003 (4)	0.004 (4)	0.004 (4)	0.004 (4)	0.004 (4)	0.003 (4)	0.003 (4)	0.003 (4)	0.003 (4)	0.003 (3)
Miscellaneous	0.001 (1)	0.001 (1)	0.001 (1)	0.001 (1)	0.001 (1)	0.002 (2)	0.002 (2)	0.001 (1)	0.000 (0)	0.001 (1)
Nucleosides and Nucleotides	0.025 (31)	0.030 (31)	0.031 (31)	0.033 (33)	0.031 (31)	0.027 (31)	0.026 (35)	0.029 (42)	0.001*** (2)	0.002 (2)
Phosphate Metabolism	0.003 (4)	0.004 (4)	0.004 (4)	0.004 (4)	0.004 (4)	0.003 (4)	0.005 (7)	0.003 (4)	0.000 (0)	0.000 (0)
Prokaryotic cell type differentiation	0.001 (1)	0.001 (1)	0.001 (1)	0.001 (1)	0.001 (1)	0.001 (1)	0.001 (1)	0.001 (1)	0.001 (1)	0.001 (1)
Protein Fate (folding, modification, targeting, degradation)	0.024 (29)	0.028 (29)	0.029 (29)	0.029 (29)	0.029 (29)	0.024 (28)	0.024 (32)	0.020 (29)	0.016 (26)	0.028 (26)
Protein Synthesis	0.130 (160)	0.149 (153)	0.154 (153)	0.119 (118)	0.157 (159)	0.101* (117)	0.129 (170)	0.112 (162)	0.097** (153)	0.164* (151)
Respiration	0.076 (94)	0.089 (92)	0.093 (92)	0.098 (97)	0.101* (103)	0.075*** (87)	0.074 (98)	0.068 (99)	0.059 (93)	0.108* (99)
RNA Processing	0.030 (37)	0.028 (29)	0.029 (29)	0.034 (34)	0.031 (31)	0.025 (29)	0.030 (40)	0.020 (29)	0.032 (50)	0.051* (47)
Stress Response, Defense and Virulence	0.040 (49)	0.038 (39)	0.039 (39)	0.040 (40)	0.040 (41)	0.035 (40)	0.042 (56)	0.025* (36)	0.034 (54)	0.049 (45)

Comparison of genes per class, according to PATRIC classification. Values correspond with the proportion of each class based on the total CDS identified. Number of CDS within each class are inside brackets. Significant differences with regard to *E. minasensis* Cuiabá are indicated by *(*p* < 0.05), **(*p* < 0.01), and ***(*p* < 0.001). Abbreviations are as follow: EMI, *E. minasensis*; ECA, *E. canis*; ECH, *E. chaffeensis*; EMU, *E. muris*; ER, *E. ruminantium*; AC, *A. centrale*; AM, *A. marginale*; AP, *A. phagocytophilum*; RC, *R. conorii*; and RP, *R. prowazekii*. Genome accessions are available in material and methods (Section 2.8).

## Supplementary Materials

Supplementary materials can be found at https://www.mdpi.com/2076-2607/7/11/528/s1.

## References

[1] Dumler J.S., Barbet A.F., Bekker C.P., Dasch G.A., Palmer G.H., Ray S.C., Rikihisa Y., Rurangirwa F.R. (2001). Reorganization of genera in the families *Rickettsiaceae* and *Anaplasmataceae* in the order Rickettsiales: Unification of some species of *Ehrlichia* with *Anaplasma, Cowdria* with *Ehrlichia* and *Ehrlichia* with *Neorickettsia*, descriptions of six new species combinations and designation of *Ehrlichia equi* and ‘HGE agent’ as subjective synonyms of *Ehrlichia phagocytophila*. Int. J. Syst. Evol. Microbiol..

[2] Cabezas-Cruz A., Zweygarth E., Vancová M., Broniszewska M., Grubhoffer L., Passos L.M.F., Ribeiro M.F.B., Alberdi P., de la Fuente J. (2016). *Ehrlichia minasensis* sp. nov., isolated from the tick *Rhipicephalus microplus*. Int. J. Syst. Evol. Microbiol..

[3] Allsopp B.A. (2015). Heartwater—*Ehrlichia ruminantium* infection. Rev. Sci. Tech..

[4] Gondard M., Cabezas-Cruz A., Charles R.A., Vayssier-Taussat M., Albina E., Moutailler S. (2017). Ticks and Tick-Borne Pathogens of the Caribbean: Current Understanding and Future Directions for More Comprehensive Surveillance. Front. Cell. Infect. Microbiol..

[5] Gajadhar A.A., Lobanov V., Scandrett W.B., Campbell J., Al-Adhami B. (2010). A novel *Ehrlichia* genotype detected in naturally infected cattle in North America. Vet. Parasitol..

[6] Lobanov V.A., Gajadhar A.A., Al-Adhami B., Schwantje H.M. (2012). Molecular study of free-ranging mule deer and white-tailed deer from British Columbia, Canada, for evidence of *Anaplasma* spp. and *Ehrlichia* spp.. Transbound. Emerg. Dis..

[7] Cabezas-Cruz A., Zweygarth E., Ribeiro M.F., da Silveira J.A., de la Fuente J., Grubhoffer L., Valdés J.J., Passos L.M. (2012). New species of *Ehrlichia* isolated from *Rhipicephalus (Boophilus) microplus* shows an ortholog of the *E. canis* major immunogenic glycoprotein gp36 with a new sequence of tandem repeats. Parasit. Vectors.

[8] Zweygarth E., Schöl H., Lis K., Cabezas-Cruz A., Thiel C., Silaghi C., Ribeiro M.F., Passos L.M. (2013). In vitro culture of a novel genotype of *Ehrlichia* sp. from Brazil. Transbound. Emerg. Dis..

[9] Aguiar D.M., Ziliani T.F., Zhang X., Melo A.L., Braga I.A., Witter R., Freitas L.C., Rondelli A.L., Luis M.A., Sorte E.C. (2014). A novel *Ehrlichia* genotype strain distinguished by the TRP36 gene naturally infects cattle in Brazil and causes clinical manifestations associated with ehrlichiosis. Ticks Tick Borne Dis..

[10] Carvalho I.T.S., Melo A.L.T., Freitas L.C., Verçoza R.V., Alves A.S., Costa J.S., Chitarra C.S., Nakazato L., Dutra V., Pacheco R.C. (2016). Minimum infection rate of *Ehrlichia minasensis* in *Rhipicephalus microplus* and *Amblyomma sculptum* ticks in Brazil. Ticks Tick. Dis..

[11] Iweriebor B.C., Mmbaga E.J., Adegborioye A., Igwaran A., Obi L.C., Okoh A.I. (2017). Genetic profiling for *Anaplasma* and *Ehrlichia* species in ticks collected in the Eastern Cape Province of South Africa. BMC Microbiol..

[12] Cicculli V., Masse S., Capai L., de Lamballerie X., Charrel R., Falchi A. (2019). First detection of *Ehrlichia minasensis* in *Hyalomma marginatum* ticks collected from cattle in Corsica, France. Vet. Med. Sci..

[13] Rehman A., Conraths F.J., Sauter-Louis C., Krücken J., Nijhof A.M. (2019). Epidemiology of tick-borne pathogens in the semi-arid and the arid agro-ecological zones of Punjab province, Pakistan. Transbound. Emerg. Dis..

[14] Li J., Liu X., Mu J., Yu X., Fei Y., Chang J., Bi Y., Zhou Y., Ding Z., Yin R. (2019). Emergence of a Novel *Ehrlichia minasensis* Strain, Harboring the Major Immunogenic Glycoprotein trp36 with Unique Tandem Repeat and C-Terminal Region Sequences, in *Haemaphysalis hystricis* Ticks Removed from Free-Ranging Sheep in Hainan Province, China. Microorganisms.

[15] Koh F.X., Kho K.L., Kisomi M.G., Wong L.P., Bulgiba A., Tan P.E., Lim Y.A.L., Nizam Q.N.H., Panchadcharam C., Tay S.T. (2018). *Ehrlichia* and *Anaplasma* Infections: Serological Evidence and Tick Surveillance in Peninsular Malaysia. J. Med. Entomol..

[16] Hailemariam Z., Krücken J., Baumann M., Ahmed J.S., Clausen P.H., Nijhof A.M. (2017). Molecular detection of tick-borne pathogens in cattle from Southwestern Ethiopia. PLoS ONE.

[17] Cabezas-Cruz A., Zweygarth E., Aguiar D.M. (2019). *Ehrlichia minasensis*, an old demon with a new name. Ticks Tick Borne Dis..

[18] Meyer D.J., Harvey J.W. (2004). Veterinary Laboratory Medicine: Interpretation & Diagnosis.

[19] Aguiar D.M., Hagiwara M.K., Labruna M.B. (2008). In vitro isolation and molecular characterization of an *Ehrlichia canis* strain from São Paulo, Brazil. Braz. J. Microbiol..

[20] Popov V.L., Han V.C., Chen S.M., Dumler J.S., Feng H.M., Andreadis T.G., Tesh R.B., Walker D.H. (1998). Ultrastructural differentiation of the genogroups in the genus *Ehrlichia*. J. Med. Microbiol..

[21] Aguiar D.M., Araujo J.P., Nakazato L., Bard E., Cabezas-Cruz A. (2019). Complete genome sequence of an *Ehrlichia minasensis* strain isolated from cattle. Microbiol. Resour. Announc..

[22] Seemann T. (2014). Prokka: Rapid prokaryotic genome annotation. Bioinformatics.

[23] Li L., Stoeckert C.J., Roos D.S. (2003). OrthoMCL: Identification of Ortholog Groups for Eukaryotic Genomes. Genome Res..

[24] Ranwez V., Harispe S., Delsuc F., Douzery E.J.P. (2011). MACSE: Multiple Alignment of Coding Sequences Accounting for Frameshifts and Stop Codons. PLoS ONE.

[25] Guindon S., Dufayard J.F., Lefort V., Anisimova M., Hordijk W., Gascuel O. (2010). New Algorithms and Methods to Estimate Maximum-Likelihood Phylogenies: Assessing the Performance of PhyML 3.0. Syst. Biol..

[26] Gouy M., Guindon S., Gascuel O. (2010). SeaView version 4: A multiplatform graphical user interface for sequence alignment and phylogenetic tree building. Mol. Biol. Evol..

[27] Zhao Y., Jia X., Yang J., Ling Y., Zhang Z., Wu J.Y.J., Xiao J. (2014). PanGP: A tool for quickly analyzing bacterial pan-genome profile. Bioinformatics.

[28] Wattam A.R., Davis J.J., Assaf R., Boisvert S., Brettin T., Bun C., Conrad N., Dietrich E.M., Disz T., Gabbard J.L. (2017). Improvements to PATRIC, the all-bacterial Bioinformatics Database and Analysis Resource Center. Nucleic Acids Res..

[29] Aziz R.K., Bartels D., Best A.A., DeJongh M., Disz T., Edwards R.A., Formsma K., Gerdes S., Glass E.M., Kubal M. (2008). The RAST Server: Rapid annotations using subsystems technology. BMC Genom..

[30] R Core Team (2018). R: A Language and Environment for Statistical Computing.

[31] Aguiar D.M., Bayry J. (2017). Ehrlichiosis. Emerging and Re-emerging Infectious Diseases of Livestock.

[32] Harrus S., Waner T., Neer T.M., Greene C.E. (2012). *Ehrlichia canis* infection. Infectious Diseases of the Dog and Cat.

[33] Al-Adhami B., Scandrett W.B., Lobanov V.A., Gajadhar A.A. (2011). Serological cross- reactivity between *Anaplasma marginale* and an *Ehrlichia* species in naturally and experimentally infected cattle. J. Vet. Diagn. Investig..

[34] Cabezas-Cruz A., Vancová M., Zweygarth E., Ribeiro M.F., Grubhoffer L., Passos L.M. (2013). Ultrastructure of *Ehrlichia mineirensis*, a new member of the *Ehrlichia genus*. Vet. Microbiol..

[35] Cabezas-Cruz A., Valdés J.J., de la Fuente J. (2014). The glycoprotein TRP36 of *Ehrlichia* sp. UFMG-EV and related cattle pathogen *Ehrlichia* sp. UFMT-BV evolved from a highly variable clade of *E. canis* under adaptive diversifying selection. Parasit Vectors.

[36] De Fine L.H.H. (2018). Does pathogen plasticity facilitate host shifts?. PLoS Pathog..

