# Supplementary figures and images for: Isolation and Characterization of a Novel Pathogenic Strain of Ehrlichia minasensis
Source: Microorganisms. 2019 Nov 5;7(11):528. doi: 10.3390/microorganisms7110528 (PMC6921006; doi:10.3390/microorganisms7110528)

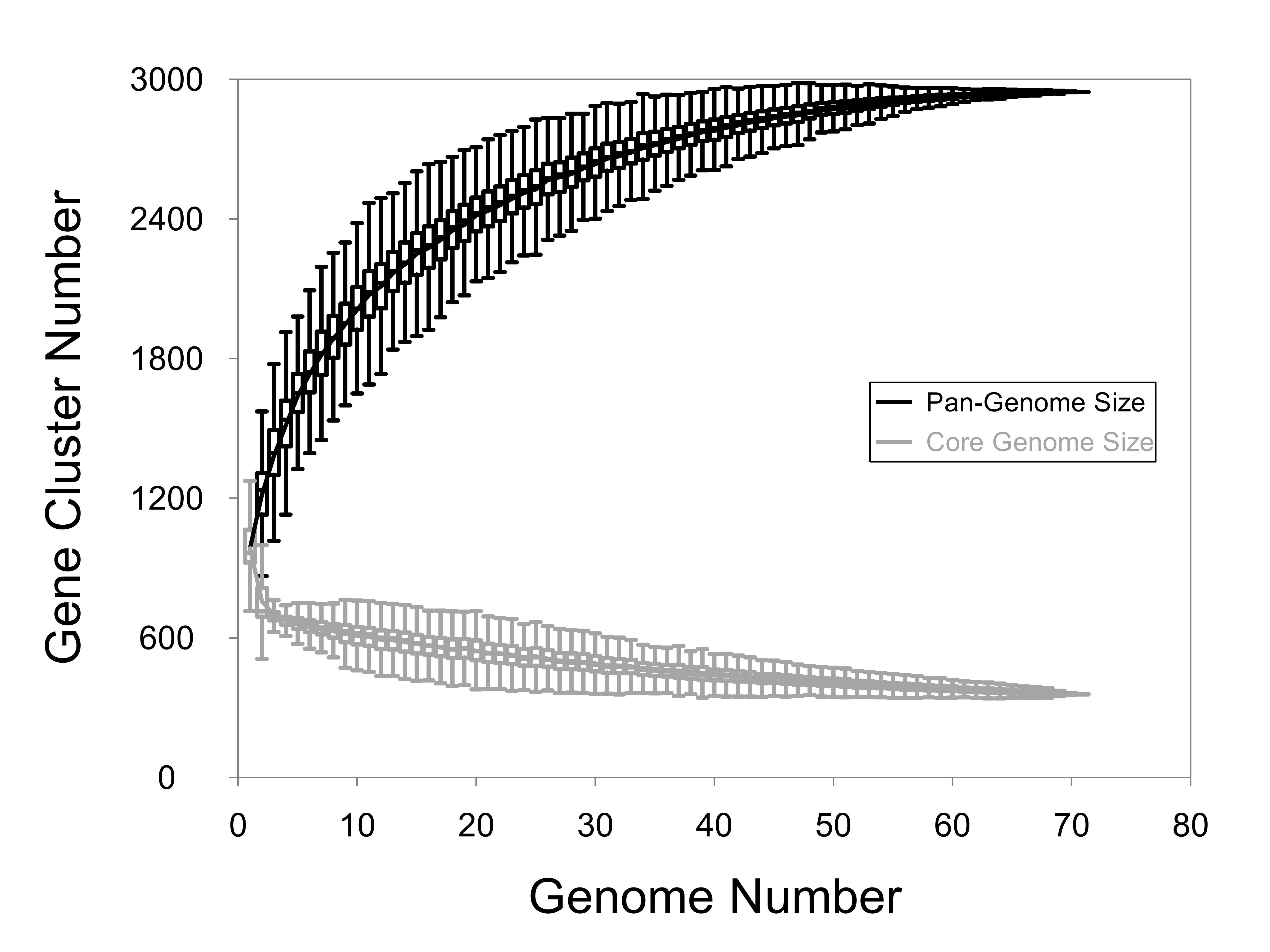

Supplement: Supplementary file 1 [file microorganisms-07-00528-s001.zip › microorganisms-623249-SI/Supplementary Figure S1.tiff]
