# Supplementary material for: Isolation and Characterization of a Novel Pathogenic Strain of Ehrlichia minasensis
Source: Microorganisms. 2019 Nov 5;7(11):528. doi: 10.3390/microorganisms7110528 (PMC6921006; doi:10.3390/microorganisms7110528)
Supplement: Supplementary file 1 [file microorganisms-07-00528-s001.zip › microorganisms-623249-SI/Supplementary Table S1.doc]

**Supplementary Table 1: *Ehrlichia* genome assemblies used in the phylogenetic tree.**

| **Bio sample ID** | **Species** | **Strain** |
| --- | --- | --- |
| **SAMN08328750** | *E. canis* | YZ-1 |
| **SAMN02598261** | *E. canis* | Jake |
| **SAMN02604010** | *E. chaffeensis* | Arkansas |
| **SAMN02203185** | *E. chaffeensis* | Heartland |
| **SAMN02364462** | *E. chaffeensis* | Jax |
| **SAMN02364461** | *E. chaffeensis* | Liberty |
| **SAMN02364994** | *E. chaffeensis* | Osceola |
| **SAMN02364995** | *E. chaffeensis* | Saint Vincent |
| **SAMN02441793** | *E. chaffeensis* | Sapulpa-1 |
| **SAMN02441793** | *E. chaffeensis* | Sapulpa-2 |
| **SAMN02367298** | *E. chaffeensis* | Wakulla |
| **SAMEA2795788** | *E. minasensis* | UFMG-EV |
| **SAMN09519635** | *E. minasensis* | Cuiabá |
| **SAMN02402773** | *E. muris* | EmCRT |
| **SAMN02641637** | *E. muris* | AS145 |
| **SAMD00050887** | *E. ruminantium* | Crystal Springs |
| **SAMEA3138254** | *E. ruminantium* | Gardel |
| **SAMD00050888** | *E. ruminantium* | Kerr Seringe |
| **SAMN04335506** | *E. ruminantium* | Palm River |
| **SAMD00050889** | *E. ruminantium* | Pokoase |
| **SAMD00050890** | *E. ruminantium* | Sankat |
| **SAMN06090168** | *E. ruminantium* | Senegal |
| **SAMN06102416** | *E. ruminantium* | Senegalp63 |
| **SAMEA1705918** | *E. ruminantium* | Welgevonden |
| **SAMEA3138255** | *E. ruminantium* | Welgevonden |
